# Structural Space of the Duffy Antigen/Receptor for Chemokines’ Intrinsically Disordered Ectodomain 1 Explored by Temperature Replica-Exchange Molecular Dynamics Simulations

**DOI:** 10.3390/ijms241713280

**Published:** 2023-08-26

**Authors:** Agata Kranjc, Tarun Jairaj Narwani, Sophie S. Abby, Alexandre G. de Brevern

**Affiliations:** 1 Université Paris Cité and Université des Antilles and Université de la Réunion, BIGR, UMR_S1134, DSIMB Team, Inserm, F-75014 Paris, France; tjrnarwani@gmail.com; 2 Institut National de la Transfusion Sanguine (INTS), F-75015 Paris, France; 3 Institute of Neuroscience and Medicine (INM-9)/Institute for Advanced Simulation (IAS-5), Forschungszentrum Jülich, D-52425 Jülich, Germany; 4 University Grenoble Alpes, CNRS, UMR 5525, VetAgro Sup, Grenoble INP, TIMC, F-38000 Grenoble, France; sophie.abby@univ-grenoble-alpes.fr

**Keywords:** chemokine receptors, molecular dynamics, *Plasmodium vivax*, malaria, protein structures, structural alphabet, entropy, rigidity, flexibility

## Abstract

*Plasmodium vivax* malaria affects 14 million people each year. Its invasion requires interactions between the parasitic Duffy-binding protein (*Pv*DBP) and the N-terminal extracellular domain (ECD1) of the host’s Duffy antigen/receptor for chemokines (DARC). ECD1 is highly flexible and intrinsically disordered, therefore it can adopt different conformations. We computationally modeled the challenging ECD1 local structure. With T-REMD simulations, we sampled its dynamic behavior and collected its most representative conformations. Our results suggest that most of the DARC ECD1 domain remains in a disordered state during the simulated time. Globular local conformations are found in the analyzed local free-energy minima. These globular conformations share an α-helix spanning residues Ser18 to Ser29 and in many cases they comprise an antiparallel β-sheet, whose β-strands are formed around residues Leu10 and Ala49. The formation of a parallel β-sheet is almost negligible. So far, progress in understanding the mechanisms forming the basis of the *P. vivax* malaria infection of reticulocytes has been hampered by experimental difficulties, along with a lack of DARC structural information. Our collection of the most probable ECD1 structural conformations will help to advance modeling of the DARC structure and to explore DARC–ECD1 interactions with a range of physiological and pathological ligands.

## 1. Introduction

The Duffy antigen/receptor for chemokines (DARC), also termed the atypical chemokine receptor 1 (ACKR1), was characterized in 1951 as the Duffy blood group antigen (Fy), being named after the patient in whom it was discovered [1,2,3,4]. The Fy gene was one of the first genes for which its location on a chromosome was precisely determined [5].

At first, the DARC receptor became well known as the most important surface receptor used by malaria parasites *Plasmodium vivax* and *Plasmodium knowlesi* to invade the host’s red blood cells (RBCs) [6,7]. It was observed that the Black African population was less susceptible to infection by *P. vivax*. Experiments by Miller et al. performed in the 1970s in American prisons showed that Fy(a-b-) individuals who did not express DARC on the RBCs, could not be infected by *P. vivax* and *P. knowlesi* [6,7]. The lack of DARC expression on the erythrocyte surface leads to the Fy(a-b-) phenotype and is caused by a single point mutation in an erythroıd regulatory element site in the DARC promoter region [8]. This mutation has a high selective value, 95% of the population in West Africa present the Fy(a-b-) phenotype that protects them against *P. vivax* and *P. knowlesi* infections [9,10,11,12,13]. It has been recently seen that the Fy(a-b-) phenotype does not ensure complete protection against *P. vivax* and *P. knowlesi* infections, suggesting that the parasites may use other ways to penetrate RBCs [14,15,16]. Nonetheless, DARC remains the major portal for *P. vivax* and *P. knowlesi* to invade the erythrocytes and, therefore, the most critical factor in malaria infections. Very recently, it was found as well, that the simian malarial parasite *Plasmodium cynomolgi* can make the zoonotic jump to infect humans as well, increasing the number of malaria parasites endangering our population [17,18]. As the closest living relative to *P. vivax*, *P. cynomolgi* also prefers to invade Fy(a+b+) individuals, using the DARC receptor to enter RBCs [19].

DARC was furthermore identified to be a chemokine receptor as well, because it was found that Fy-positive, but not Fy-null, erythrocytes bind to CXCL-8 (known also as Interleukin-8) [20,21]. CXCL8 is implicated in conventional chemotaxis. Chemokine receptors are a large family of seven segment transmembrane proteins coupled with G-proteins [22,23]. They are implicated in a large spectrum of essential biological activities, such as homeostatic and inflammatory processes [24,25]. They bind two main families of chemokines, namely CCL and CXCL, defined this way as the first two Cys residues are adjacent or separated by one amino acid, respectively. Two other, minor and quite specific, types of chemokines exist: CL chemokines with only one Cys close to the N-terminus and CX_3_CL chemokines, where three amino acids separate the first two Cys residues [22]. A chemokine ligand binds to a specific chemokine receptor, triggering cell signalling through the activation of G-proteins via the classical phospholipase C pathways. Chemokine receptors are named according to the type of chemokines they bind to: CC receptors bind specifically to CCL chemokines and CXC receptors bind to CXCL chemokines, and so on. DARC, however, binds promiscuously to both CCL and CXCL chemokines and does not signal downstream. Hence, it is also called an atypical chemokine receptor 1 (ACKR1) or the silent receptor. It binds a large variety of chemokines: CCL-2, CCL-5 (previously named RANTES), CCL-11, CCL-13, CCL-14, CCL-17, CXCL-1, CXCL-4, CXCL-5, CXCL-6, CXCL-7, CXCL-8 and CXCL-11. DARC is a silent receptor due to the absence of the highly conserved DRY (Asp–Arg–Tyr) motif in the second intracellular loop of the protein that is associated with G-protein signalling. Hence, DARC cannot provide any protein-coupled signal transduction or a Ca^2+^ flux [26,27,28,29,30].

As for most of the transmembrane proteins, the DARC experimental 3D structure is unknown. A preliminary structural model was generated more than a decade ago and was assessed in regard to 40 point mutations known to affect the binding of CXCL-8 to DARC [31,32]. DARC clearly has a topology related to all other members of chemokine receptors, with a large N-terminus region before the first transmembrane α-helix. This extracellular N-terminal region is composed of more than 60 residues and named the extracellular domain 1 (ECD1). ECD1 is directly implicated in the binding of the *P. vivax* Duffy binding-like domain (DBL) [27,28,33,34], enabling *P. vivax* parasites to enter the erythrocytes, causing blood stage infection and triggering all the clinical symptoms of malaria. Therefore, one of the main therapeutic strategies is to prevent the interaction between the DARC–ECD1 domain and the *P. vivax* DBL domain, as this could alleviate malarial disease.

ECD1 is highly flexible, and its intrinsically disordered nature was confirmed by an attempt to solve a co-crystal structure of *P. vivax* Duffy binding-like domain (DBL) together with ECD1. But, for the latter, only a nine residues long peptide Gln19–Tyr30, an α-helix, was successfully solved (PDB ids: 4NUU and 4NUV) [35]. The characteristic feature of such domains is that they can adopt many different conformations. The difficulties in obtaining its experimental structure limits further computational studies and analyses. Therefore, in our work we constructed a relevant structural model of ECD1 and explored its conformational space with the enhanced sampling technique T-REMD (temperature replica-exchange molecular dynamics). Our extensive analyses of ECD1 conformations provides an important piece of knowledge for the molecular modeling and computational drug design communities.

## 2. Results

### 2.1. DARC–ECD1 Structural Model

Structural models of ECD1 were built by means of de novo modeling. Twenty different ECD1 structural models were selected, five from each structure prediction server (ROBETTA [36], I-TASSER [37], QUARK [38] and LOMETS [39]).

To compare the different models, we opted for structural clustering using the protein blocks method [40,41]. But, due to the disordered nature of the ECD1 domain, no real clustering could be observed. Please note that the AlphaFold2 [42,43] model was also analyzed and, as expected [44,45,46], it did not provide any new insights on the local conformations.

**Figure 1:**
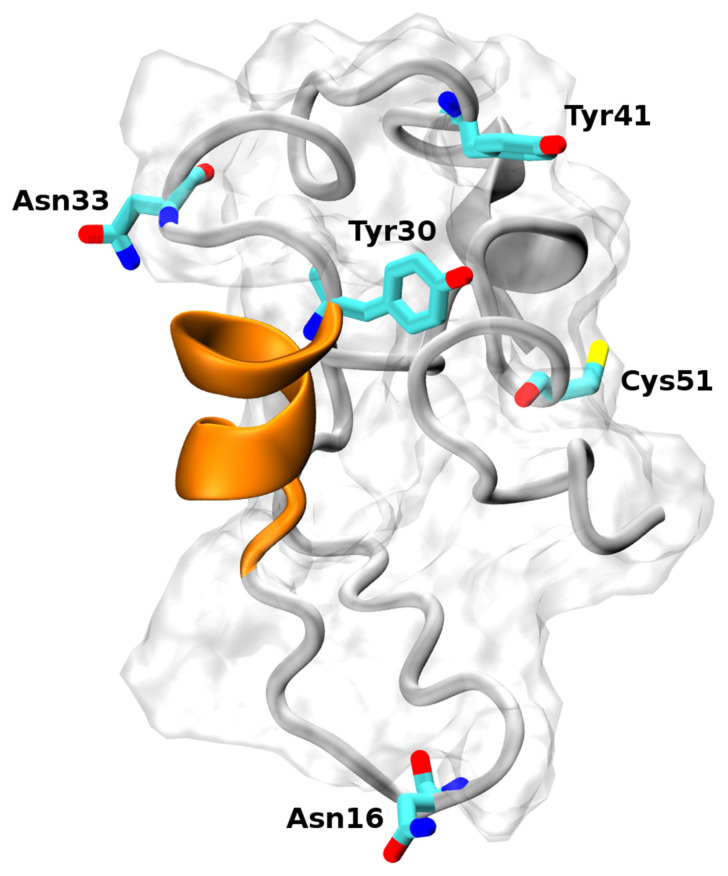
Structural model of the DARC–ECD1 domain. The α-helix spanning residues Phe22–Tyr30 is highlighted in orange. The two tyrosines (positions 30 and 41) can be sulfated and are important for the binding of the *Plasmodium vivax* Duffy binding protein. The asparagines (positions 16 and 33) can be glycosylated. The Cys51 residue forms a disulphide bridge with Cys276, located in the 4th extracellular loop in the DARC transmembrane region.

Hence, only experimental bibliographic information was used to select the most relevant models. Firstly, we assessed the models for the presence of the α-helix spanning residues Phe22–Tyr30, as was found in the crystallographic study [35]. Secondly, we looked for the solvent accessibility [47] of the residues involved in the post-translational modifications and in the inter-/intra-molecular binding. Indeed, ECD1 undergoes different post-translational modifications, like the glycosylation of Asn16 and Asn33 [48,49] and the sulfation of Tyr30 and Tyr41 [34]. Sulfated tyrosines are crucial for the binding of chemokines and of *Pv*DBP [34]. Finally, we considered the solvent accessibility of Cys51 that should form a disulphide bond with Cys276, present in the fourth extracellular loop in the DARC transmembrane region. One model (see Figure 1), generated by the Quark prediction server, satisfied all of these constraints, and was further studied with temperature replica-exchange molecular dynamics (T-REMD) simulations.

### 2.2. Analysis of the T-REMD Simulations

The selected model was subjected to T-REMD simulations for a total simulation time of 12.8 μs in the temperature range of 310–400 K; the replica at the lowest temperature was analyzed. Throughout the MD simulations, ECD1 adopts a large variety of conformations from very extended to more compact ones. But, even in the compact/globular conformations, most of the sequence length remained unstructured, featuring long and flexible loops.

**Figure 2:**
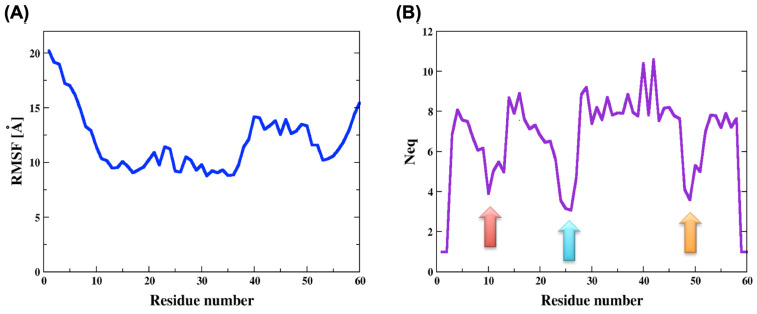
Analysis of the molecular dynamics simulations of ECD1. (**A**) The root mean square fluctuations (RMSf) and (**B**) the equivalent number of protein blocks (Neq) were calculated for each residue of the ECD1 domains. The lowest *N_eq_* values are indicated by the three colored arrows.

The root mean square fluctuation (RMSf) values were very high and as expected the N and C termini had higher values than the core of the domain. Nevertheless, the RMSf of the two sections, containing residues 10 to 35 and residues 50 to 55 were a bit lower with respect to the rest of the chain (see Figure 2A). Lower flexibility, though still very high, can indicate a possibility for secondary structures formation or other intramolecular interactions.

To understand more in detail about the local and global structural composition of ECD1, we performed analyses using protein blocks (PBs) and collective variables for secondary and tertiary structures analyses, respectively.

### 2.3. Local Structural Analysis of ECD1

By means of protein blocks (PBxplore tool [41]), we calculated the PBs occurrence, i.e., the local conformation of each residue in the ECD1 chain throughout the MD run. In addition, we calculated the *N_eq_* (the equivalent number of PBs), which is a statistical measurement quantifying the average number of PBs a residue at a given position can adopt (Figure 2B, see Methods for more details). Low *N_eq_* values means that the residue is assigned to a few different PBs, its dihedral angle connecting it to the neighboring residues varies only slightly and the residue fluctuations are low. Accordingly, the *N_eq_* tells us which sections of a protein chain are more structured. We noticed that the *N_eq_* greatly correlates with the RMSf, where the former is a local, and the latter is a global, measure of a protein’s flexibility.

The lowest RMSf values were observed for the region of residues 10 to 35, and one of the lowest *N_eq_* values was around residue 25 (see the blue arrow in Figure 2B). They both indicate higher structural stability, corresponding very well to the fact that the residues Phe22 to Tyr30 form an α-helix, as it was found when ECD1 is bound to the *P. vivax* Duffy binding protein [35]. 

**Figure 3:**
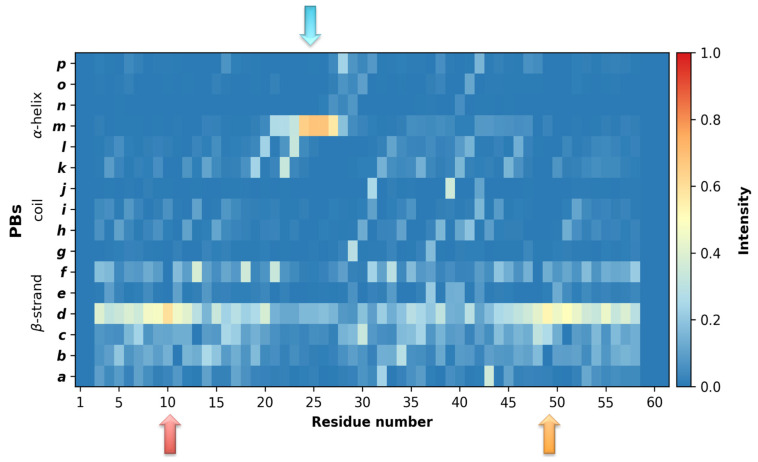
Analysis of the ECD1 dynamics in light of the protein blocks (PBs). Occurrence map for the PBs, observed during the simulations, providing a reliable association with the secondary structures. The x-axis represents the residue positions and the y-axis represents the 16 PBs, ranging from PB *a* to PB *p*. The colors correspond to the occurrence frequency, ranging from blue (0, never observed) to red (1.0, always observed). The lowest *N_eq_* values are pointed out by the arrows.

Analyses of the MD simulations, in light of the PB distribution, confirmed a high probability for an α-helix formation at this position, not only when ECD1 is bound to its partner protein, as shown by the experiments, but also when it is isolated, as shown in this study. In fact, during the MD simulations, ECD1 residues Glu23 to Asn27 exhibited a ~70% probability of forming an α-helix (see the blue arrow in Figure 3). The high stability of this α-helix confirms its importance as the epitope for the *P. vivax*, *P. knowlesi* and *P. cynomolgi* invasion. In addition, in the rest of the ECD1 chain, the probability of α-helix formation is null, confirming its emplacement in the ECD1 chain. 

Furthermore, the *N_eq_* graph clearly shows a tendency for secondary structure formation around the residues 10 and 49 (see red and orange arrows in Figure 2B). Strikingly, the protein blocks analyses show that in these regions there is ~50% probability for β-strands formation (see the same arrow positions in Figure 3). 

From the PBs analyses, it is clear that ECD1 has well-defined secondary structure preferences at well-defined residue positions. The remaining questions concern the dynamics of these local protein conformations. Do β-strands arrange in β-sheets? Is an α-helix formed simultaneously with β-sheets? And, most importantly, is there a large number of local conformations and, if yes, then how do they organize?

To answer these questions, we projected the trajectories onto collective variables, able to quantify the content of the secondary and tertiary structure [50], and we traced the resulting free-energy surface (FES; see Methods for details). In this way we clearly display the correlations between the secondary structures (α-helices and β-strands) and the radius of gyration (for the latter see Figure S1), and we find different ECD1 conformational representatives.

### 2.4. Global Structural Analysis of ECD1

#### 2.4.1. Analysis of the α-Helix Content

**Figure 4:**
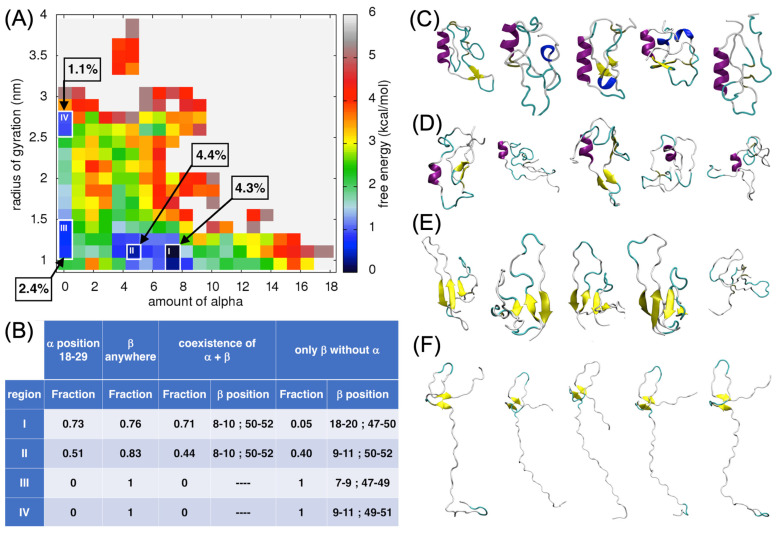
Analysis of the α-helix content in ECD1. (**A**) Free-energy surface (FES) plot as a function of the amount of α-helices vs. the radius of gyration. The four regions, i.e., the local minima I–IV, that were analyzed more in detail are shown and the fraction of the structures found in each of them are indicated. On the right, the color code bar on the estimated free-energy values is provided (the white space represents non-populated values). (**B**) The fraction of the ECD1 conformations in the regions I–IV that have: (I) an α-helix at positions 18–29, (II) a β-strand formed anywhere in the sequence, (III) an α-helix at positions 18–29 and at the same time as the β-strands; the range of the residue positions having the highest probability to form a β-strand are reported, (IV) an α-helix at positions 18–29 is absent and only β-strands are present; the range of residue positions having the highest probability of forming β-strands in this case are reported. (**C**–**F**) 3D representations of the representative ECD1 conformations from the five most populated clusters found in regions I–IV, respectively. See also Data S1.

We plotted the free-energy surface for the amount of α-helices versus the radius of gyration (Rg; Figure 4A). Four distinct local minima are visible in the plot (regions I–IV; see their localization in Figure 4A, their characteristics in Figure 4B and Table S1, and the 3D representatives in Figure 4C–F). Region I represents the deepest local minimum, where the amount of α-helices is around seven and the value of the Rg is around 1 nm. Region II has a shallower local minimum compared to that of region I, structures found in this minimum have around four α-helices and the radius of gyration is around 1 nm. About 4% of all the structural conformations formed during the MD simulations satisfied the criteria of regions I and II. The ECD1 conformation representatives from these two regions have an α-helix of differing lengths, but always formed in the same position, between residues Ser18–Ser29. In region I, 73% of the ECD1 conformations have an α-helix in this position, while in region II, it is 51% (Figure 4B). 

Two β-strands formed at the positions 1–20 and 40–60 and were assembled into an antiparallel β-sheet. Overall, 71% of the conformations found in region I were composed of one α-helix and at the same time as the antiparallel β-sheet, at the abovementioned positions. In region II, the α-helix and β-sheet co-existed in 44% of the conformations. The β-strands constituting the β-sheet were again formed in the positions 1–20 and 40–60 (Figure 4B). The conformations within regions I and II were globular, as expected from their low radius of gyration, despite that the majority of the ECD1 chain remained as random coil conformations, as expected for an IDR.

In regions III and IV, the amount of α-helices was 0 and the Rg was around 1 nm and 2.5 nm, respectively. Overall, 1% and 2% of all ECD1 MD conformations were found in these two regions, respectively. These conformations were of interest as they have no α-helices, but were composed of two- to four-stranded antiparallel β-sheets, or exclusively of random coils. Hence, ECD1 can adopt, as well as compact, conformations in the absence of the α-helix, where four-stranded β-sheets are present (region III; Figure 4E). However, more extended conformations are favored when only two-stranded β-sheets are formed (as in region IV; Figure 4F).

#### 2.4.2. Analysis of the Antiparallel β-Sheet Content

**Figure 5:**
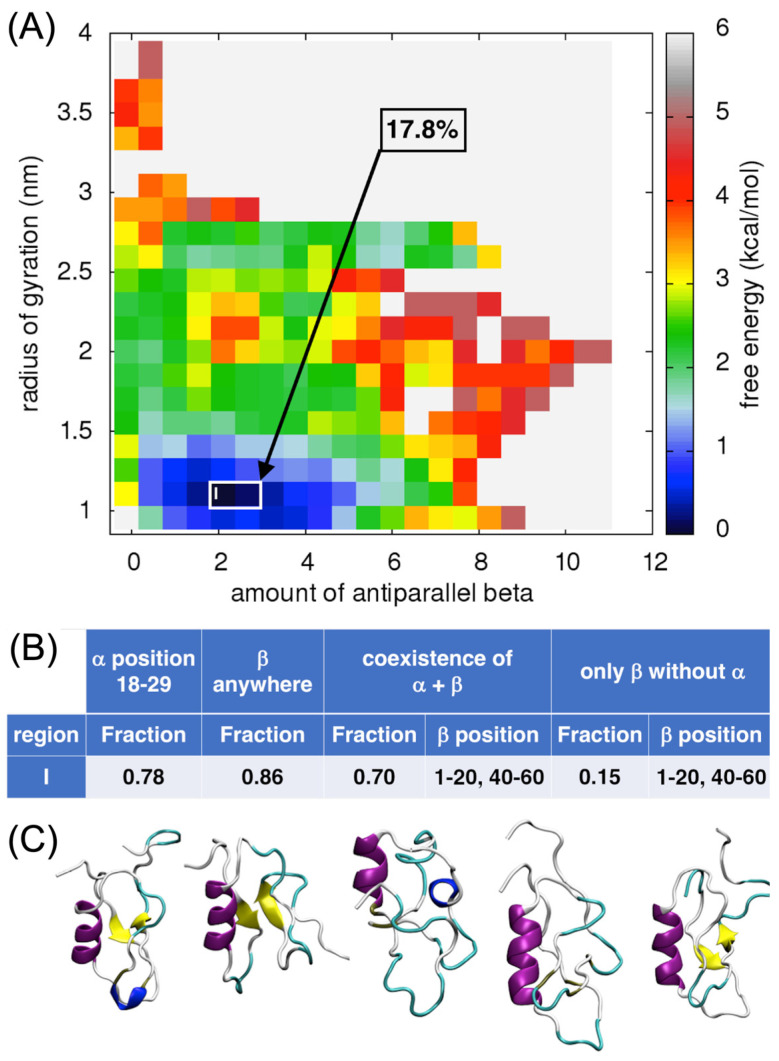
Analysis of the antiparallel β-sheet content in ECD1. (**A**) FES plot as a function of the amount of antiparallel β-sheets vs. the radius of gyration. (**B**) The fraction of the ECD1 conformations. (**C**) 3D representations of the representative ECD1 conformations from the five most populated clusters found. See also Data S2.

In the case of the antiparallel β-sheet content, one well-defined local minimum was found for an amount of antiparallel β-sheet of 2.5 and the value of the Rg was around 1 nm (region I, Figure 5A). About 18% of all the ECD1 conformations from the MD run corresponded to these criteria. It is important to note that all five major representative conformations do have an α-helix, encompassing residues between Ser18 and Ser29. Indeed, the α-helix at this position is present in 78% of the conformations found in this local minimum (Figure 5B,C). In 70% of the conformations, an antiparallel β-sheet, always including residues at positions 1–20 and 40–60, coexists with the α-helix (Figure 5B,C). These results agree with the protein blocks analyses (see Figure 1, Figure 2B and Figure 3). 

Counter-intuitively, the third and the fourth conformations in Figure 5C do not display β-strands. This can be understood considering the mathematical definition of the β-sheet collective variables, that is the sum over the contributions, each one scoring between zero and one. For instance, the total value of one can be the result of two locations contributing one half (hence, with only a partially-formed β-sheet structure at each location), as well as of a single location contributing a perfectly formed β-sheet unit. The presence or absence of β-strands in the visualization depends on the rules implemented by STRIDE [51] in the VMD program [52]. Indeed, as we have shown in multiple analyses, the secondary structure assignment depends on many parameters and often two different secondary structure assignment approaches assigned differently ~20% of the residues [53,54,55]. To check this phenomenon, PB [41] and DSSP analyses were performed. Both analyses concurred that residues attributed to form β-strands in the conformation, at the positions Gln19–Asp21 and Asn44–Glu46. For the fourth conformation, the DSSP analysis did not assign β-strands, while PBxplore found two well-defined β-strands in the positions Asn3–Pro12 and Leu45–Ser53 (Figure S2), even though it did not provide information about the β-strands orientation, parallel or antiparallel. This indicates that the lack of β-structures in the VMD could rather be an artifact of visualization. 

#### 2.4.3. Analysis of the Parallel β-Sheet Content

**Figure 6:**
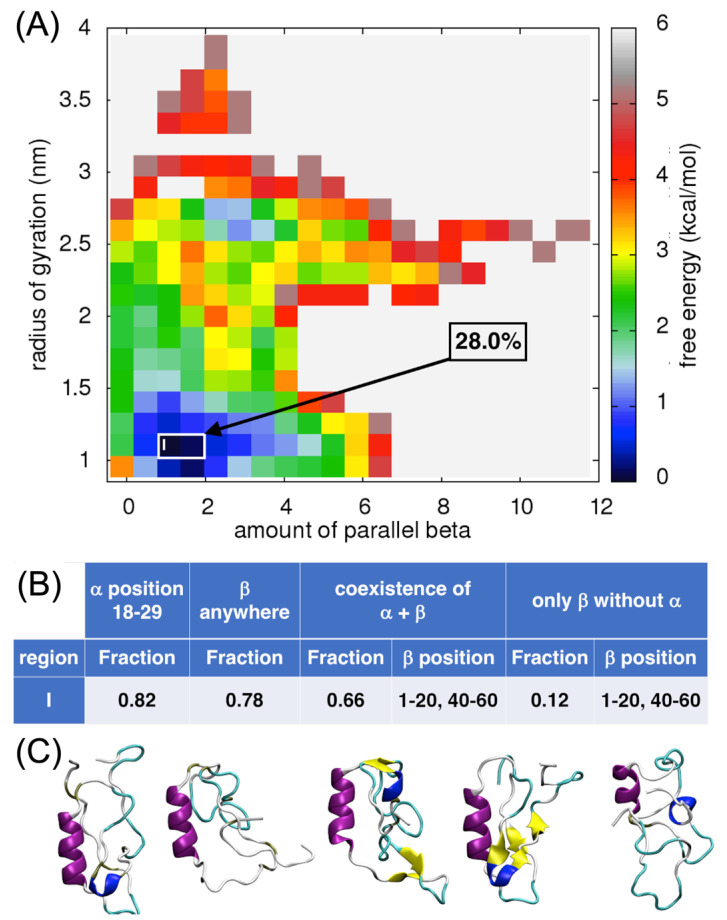
Analysis of the parallel β-sheet content in ECD1. (**A**) FES plot as a function of the amount of parallel β-sheets vs. the radius of gyration. (**B**) The fraction of the ECD1 conformations. (**C**) 3D representations of the representative ECD1 conformations from the five most populated clusters found. See also Data S3.

Analysis of the free-energy surface for the amount of parallel β-sheet versus the radius of gyration showed the lowest local minimum when the amount of parallel β-sheets is between 0.6–2.0 and the radius of gyration is about 1.0 nm (Figure 6A). Overall, 28% of the ECD1 conformations from the MD simulations correspond to these criteria. Here, again, it is worth noting, that an α-helix is present in 82% of the selected conformations, involving residues Ser18–Ser29 (Figure 6B,C) and, in 66% of the cases, the α-helix co-exists with a β-sheet. None of the selected conformations have parallel β-sheets, but rather antiparallel β-sheets. Indeed, the total amount of parallel β-sheet contributions to the local minimum is very low (0.6–2.0) and corresponds to several incomplete β-sheet units rather than to one or two well-formed ones. To investigate further the formation of β-strands and β-sheets we used DSSP and STRIDE tools. Both tools are in very good agreement concerning the assignment of the secondary structure and found β-strands in representative structures three and four (as shown in Figure 6C), while in other conformations they assigned only a β-bridge conformation for up to six residues sparsely around the ECD1 chain. While the STRIDE output does not distinguish between the parallel and antiparallel β-sheet conformations, the DSSP does, and it recognized all the β-sheet conformations as antiparallel and none as parallel. 

Comparing the FES plots in Figure 5A and Figure 6A, one can see that in the respective lowest local minima the amount of antiparallel β-sheets (~2.5) is higher than the amount of parallel β-sheets (~1.5). A comparison of the ECD1 structural conformations in Figure 4C–F, Figure 5C and Figure 6C show that all the β-structural elements are antiparallel β-sheets and none are parallel. Based on these results, we suggest that antiparallel β-sheets are more favorable and more probable structural elements in the ECD1 domain than parallel β-sheets. 

#### 2.4.4. Analysis of the α-Helix and the Antiparallel β-Sheet Content

**Figure 7:**
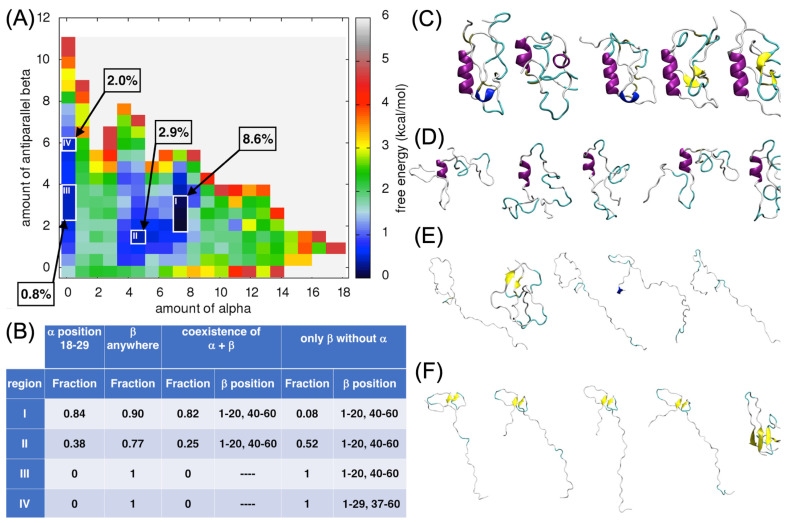
Analysis for the α-helices versus the antiparallel β-sheet content in ECD1. (**A**) FES plot as a function of the amount of α-helices vs. the amount of antiparallel β-sheets. (**B**) The fraction of the ECD1 conformations in the regions I–IV that have: (I) an α-helix at positions 18–29, (II) a β-strand formed anywhere in the sequence, (III) an α-helix at positions 18–29 and at the same time as the β-strands; the range of the residue positions having the highest probability to form a β-strand are reported, (IV) an α-helix at positions 18–29 is absent and only β-strands are present; the range of residue positions having the highest probability of forming β-strands in this case are reported. (**C**–**F**) 3D representations of the representative ECD1 conformations from the five most populated clusters found in regions I–IV, respectively. See also Data S4.

Finally, the FES for the amount of α-helices versus the amount of antiparallel β-sheets was analyzed, underlying four different regions corresponding to the various local minima (regions I to IV; Figure 7A). The deepest local minimum corresponded to the amount of α-helix of around 7.5 and to the amount of antiparallel β-sheet of around 2.5. More than 8% of all the ECD1 conformations, from the MD run, were found in this local minimum (region I, Figure 7A). 

The ECD1 structural conformations found in region I are compact/globular and 84% of them have an α-helix spanning between the residues Ser18–Ser28 (Figure 7B,C). The antiparallel β-sheets are formed most often at the positions 1–20 and 40–60, and they co-exist with the α-helix in 82% of the selected conformations (Figure 7B,C). 

In region II, the amount of α-helices is around 4.5 and the amount of antiparallel β-sheets is low, at around 1.5. About 3% of all the ECD1 conformations obtained during our MD simulations were found in this local minimum. Overall, 38% of these conformations have α-helix encompassing residues Ser18–Ser28. In 25% of the conformations belonging to this region, the antiparallel β-sheets co-exist with the α-helix (Figure 7B,D). 

In the regions III and IV, the amount of α-helices is zero, but the amount of β-sheets is around three and six, respectively. In region III, less than 0.8% of the ECD1 conformations were found, while region IV comprised 2% of them (Figure 7A). According to our calculations, the ECD1 conformations found in regions III and IV have 100% probability that a β-strand is formed within the chain (Figure 7B). However, in region III, the amount of beta sheets is low (values 2–4, Figure 7A). Consequently, there is substantial probability that only single beta strands are formed. In region III, representative conformations are mostly extended, forming a random coil state; only one representative structure has a small antiparallel β-sheet with proper hydrogen bonds (Figure 7E). The calculations on the probability of the secondary structure formation, presented in Figure 7B, were conducted based on the PB assignment of the secondary structure conformation. We found that β-strands can be formed if there are at least three consecutive residues assigned to the beta conformation, and the result is that this is the case in all the ECD1 conformations found in regions III and IV. Missing β-strands in the representative structures in region III are most probably an artifact of the VMD software (version 1.9.4), as already discussed above. The only conformation in region III that has a β-sheet is quite compact, though this is not always the case when β-sheets are formed, as it can be seen for the most of the conformations in region IV (Figure 7F), as well as for the conformations in region IV in Figure 4F.

As for region III, also in region IV, most of the conformations are extended, but due to the higher amount of β-sheets (value six on the vertical axis, Figure 7A), this motif is observed in all five representative structures (Figure 7F). One of the structures is more compact, comprising of four antiparallel β-strands connected by long loops composed of 13–15 residues. The highest probability for β-strand formation in the ECD1 conformations belonging to this region is at the positions 1–20, 21–40, and 40–60 (Figure 7B,F).

Taken together, the analyses of all the FES plots show that conformations with only β-strands can exist, but are less favorable (i.e., the local free-energy minimum is shallow) than the conformations with an α-helix.

## 3. Discussion

DARC is a transmembrane protein, whose extracellular disordered region (ECD1) is essential for the binding of numerous chemokines belonging to the CC and CXC chemokine families [56]. Intrinsically disordered regions are common in transmembrane proteins, though occurring more often in the intracellular than extracellular domains [57,58]. It is argued that the flexibility of these regions is important for the binding of different ligands. Indeed, ECD1, beside binding promiscuously to two different classes of chemokines, is also the major binding partner for Duffy binding proteins from *P. vivax*, *P. knowlesi* and *P. cynomolgi*, facilitating or enabling the entry of these parasites into RBCs and triggering malarial infection [6,7,17]. The DARC protein remains, up to today, structurally unresolved, however some attempts have been made to model it [32] and to resolve at least its ECD1 in a complex with the receptor binding domain of the *P. vivax* Duffy binding protein; however, for ECD1, only a short α-helix, spanning the residues of Phe22–Tyr30, could be successfully resolved [35].

The main scope of our work was to explore, exhaustively and comprehensively, the conformational space of ECD1, in order to identify its most probable conformations that can further serve to complete the DARC structural model of the transmembrane domain. This would allow detailed in silico studies of complexes between DARC and its partners. We also provide the scientific community with the publicly available set of DARC–ECD1 conformations for further studies.

To attain the scope, the ECD1 domain had to be modeled and this proved to be challenging for two reasons. Firstly, no template with adequate sequence similarity to ECD1 could be identified. The phylogenetic analyses performed in our group revealed that the closest homolog in the evolutionary tree was the G-protein coupled receptor 35 (GPCR35), but none of the members in this clade were a chemokine receptor, all of them were 7-transmembrane GPCRs. The closest chemokine receptor homologs were ACKR3 members, but the 3D structures for these receptors had not yet been resolved when this work was carried out. The homologous chemokine receptor families with resolved 3D structures are CXCR4 receptors [59]. However, both ACKR3 and CXCR4 are very distant phylogenetically from DARC (ACKR1), with very different ECD1s [59,60]. Secondly, ECD1 is intrinsically disordered and, therefore, may adopt many different conformations, including changes in the secondary structure formation. To overcome these bottlenecks, complex de novo modeling was performed to propose the ECD1 structural model employing different modeling servers available at the time of the analysis and including all the available experimental data for DARC–ECD1. As previously mentioned, the new AlphaFold2 methodology [42,43,61] does not provide new insights on ECD1 conformations. Similarly, at the time of the writing, the structure of DARC was deposited on bioRxiv [62]. Authors have determined its cryo-EM structure in a complex with CCL7 revealing a relatively superficial binding mode of CCL7, but do not provide new insights on ECD1 dynamics [62].

In the second step of the ECD1 structural predictions, the enhanced sampling technique (T-REMD) was used to explore ECD1’s structural conformational space. T-REMD is an effective method that improves sampling in MD simulations of biomolecular systems, by simulating replicas of the system at a range of different temperatures and periodically exchanging between them [63]. This type of simulation allowed us to better explore ECD1’s dynamical behavior, to sample its fluctuations and to select the most representative conformations, i.e., to define the possibilities that ECD1 adopts more globular forms, and to identify the potential regular secondary structures.

By means of PBs, we then determined the local probability for secondary structures formation and by using FES we identified the probabilities for the global secondary and tertiary structural architecture of ECD1. The PBs analysis, that includes the full-length MD trajectory (i.e., 40,000 ECD1 conformations), underlines that residues Glu23 to Asn27 possess a probability of about 70% to form an α-helix and that β-strands have about 50% probability to be formed at the N-terminal part of the ECD1, around residue Leu10, and at the C-terminal part of the domain, around residue Ala49 (Figure 3). 

Furthermore, the FES analyses allowed us to select and focus on the globular conformations found in well-defined local minima. Based on our T-REMD results, most of the DARC–ECD1 chain is disordered, associated with a random coil state with no secondary structures formation. The globular forms are thermodynamically favored, i.e., they correspond to the lowest free-energy regions in the FES landscapes (regions I–IV in Figure 4A, Figure 5A, Figure 6A and Figure 7A).

From 73% to 84% of the globular ECD1 conformations found in the lowest free-energy minima have an α-helix in the position between Ser18 to Ser29 (see regions I in Figure 4B, Figure 5B, Figure 6B and Figure 7B). In addition, 76% to 90% of these conformations have a β-strand formed anywhere in the chain. Furthermore, the ECD1 conformations found in these regions have 66% to 82% probability that an α-helix at the position Ser18 to Ser29 co-exists with a β-strand. After careful examination, we see that when the α-helix and the β-strand secondary structures co-exist, the latter are most of the time in the form of an anti-parallel β-sheet and the highest probability for the formation of the two β-strands are in the N- and C-terminal regions of the ECD1, comprising residues 1–20 and 40–60, respectively (Figure S2A–D, region I, and Table S1, region I).

In Figure 4 and Figure 7, region II corresponds to the local minima that are energetically less favorable and have a lower amount of α-helices than the local minima in region I. With respect to region I, less conformations (51% and 38%, respectively) have an α-helix at the position Ser18 to Ser29 and, as expected, based on the lower amount of α-helix, they are shorter. The probability that the α-helix and β-strand(s) co-exist is much lower with respect to region I, while the fraction of conformations encompassing only β-sheets is much higher than in the same regions. Interestingly, β-strands are still favorably formed at the N- and C-terminal positions 1–20 and 40–60 (Figure S3A,D, region II, and Table S1, region II).

Furthermore, we analyzed other distinct local minima, corresponding to regions III and IV in Figure 4 and Figure 7. In these regions, the amount of α-helix is zero, meaning that they include only the conformations with β-strands and/or random coil forms. The β-strands still form preferably at the N- and C-terminal positions 1–20 and 40–60, but the local probability for beta formation is high also at positions in the range 20–40 (Figure S4A–D, regions III and IV, Table S1, regions III and IV).

Our results indicate that in ECD1 conformations, antiparallel β-sheets are far more probable than parallel β-sheets. Indeed, in the analyses performed specifically for parallel β-sheets (Figure 6A), we did not observe the formation of the latter in any of the selected representative conformations. However, conformations with antiparallel β-sheets were observed (Figure 6C), indicating that the amount of antiparallel β-sheets is higher than the amount of parallel β-sheets.

## 4. Materials and Methods

### 4.1. Structural Modelling of ECD1

The human DARC (ACKR1) sequence corresponds to UniProt entry Q16570. ECD1 was defined as the first 60 residues. De novo modelling was used to build DARC–ECD1 structural models by means of four different state-of-the-art structure prediction servers, namely ROBETTA [36], I-TASSER [37], QUARK [38] and LOMETS [39], and the recent AlphaFold2 algorithm [42,43]. The ECD1 structural models were analyzed with the protein blocks method (see below). The selection of the best structural model was conducted based on the knowledge, based on integrated information from crystallographic data and experimental data, that certain residues must be highly accessible to solvent in order to interact with other proteins or DARC domains.

### 4.2. Temperature Replica-Exchange Molecular Dynamics (T-REMD)

All molecular dynamics (MD) simulations were performed with GROMACS 2016.4 [64], locally and on the supercomputer Occigen at the CINES computing center in France. The structural model of ECD1 was inserted into a box filled with ~7000 water molecules, 9 Na^+^ counter ions were added to ensure systems charge neutrality. The force field for the protein, ions and water was CHARMM36m [65] with TIP3P water molecules.

The initial geometry of the ECD1 model was optimized by the steepest descent minimization performed for 50,000 steps, with a maximum force constant value of 10 kJ/mol/nm. After the geometrical optimization, the NVT equilibration was run for 100 ps with a time step of 2 fs and the target temperature was set to 310 K. Then, the system underwent NPT equilibration for 50 ns, with a time step of 2 fs. The system was maintained at the reference pressure of 1 bar and a temperature of 310 K, by coupling to the Berendsen barostat and stochastic velocity rescaling thermostat [66], respectively. The position restraints on the ECD1 domain corresponded to a harmonic force constant of 1000 kJ mol^−1^ nm^−2^.

Finally, the unrestrained replica-exchange simulations were performed with 32 replicas in the temperature range between 310 K and 400 K. For each replica, the system was first equilibrated for 1 ns at the target temperature, then the MD simulation was run for 400 ns (total time of 12.8 μs = 32 × 400 ns), attempting exchanges every 2 ps. The temperature distribution was obtained through an online temperature generator for REMD simulations [67], giving equally-spaced temperatures with steps of 2.903 K (i.e., T*_i_* = 310 + 2.903 × *i*, *i* = 0…31). The system was coupled to the same barostat and thermostat as during the NPT equilibration.

A time step of 2 fs was used. All bond lengths were kept fixed applying the LINCS algorithm [68]. Periodic boundary conditions were applied, treating long-range electrostatic interactions with the particle-mesh Ewald technique, using a short-range cut-off of 1.2 nm [69]. The same cut-off was used for the van der Waals interactions.

### 4.3. Analysis of Statistical Convergence Based on RMSD Clustering

The analyses were performed with the replica at *T* = 310 K. The convergence of the replica exchange simulations was assessed by cluster analysis. The cluster centers were defined upon a trajectory with the GROMOS clustering algorithm, as implemented in GROMACS. This iterative approach takes cut-offs ranging between 1.0 Å and 3.5 Å with a step of 0.5 Å. It used the backbone RMSD and omitted the C- and N-termini, as they are always flexible, i.e., the analyzed system corresponds to residues 8 to 55. Then, all the configurations along the trajectory were assigned to the nearest cluster center using Voronoï-like partitioning.

Comparing the cluster populations in two segments of the 310 K trajectory tested the convergence of the T-REMD: between 0–200 ns and 200–400 ns, and between 100–250 ns and 250–400 ns. In the latter case, the first 100 ns were discarded as the initial exploration of the conformational landscape.

At convergence, the trajectory must explore ergodically all the clusters in a repeated way, leading to stationary (equilibrium, Boltzmann-like) populations when estimated over sufficient durations. Our analyses showed that this is the case for some of the clusters, but not for most of them, meaning that the system has not yet reached convergence. To reach convergence using the T-REMD is challenging per se, and in our case this challenge is increased by the fact that we are dealing with an intrinsically disordered domain. We think that looking for a convergence using RMSD clustering is not the most appropriate way for intrinsically disordered regions, therefore we rather evaluated the convergence with secondary and tertiary structures variables (see below) [50]. 

### 4.4. Protein Blocks

Protein blocks (PBs) is a widely used structural alphabet composed of 16 local prototypes [40]. It is employed to analyze local conformations of protein structures from the Protein Data Bank (PDB) [70]. Each PB is characterized by the φ and ψ dihedral angles of five consecutive residues. PBs provide a reasonable approximation of all the local protein 3D structures [71], with a median root mean square deviation of 0.34 Å, a median root mean square deviation on angular values (rmsda) of 26.1° and a mda_120_ of 98.8% [72]. Some PBs have better approximation than others, ranging from PB *m* (median rmsda of 7.6°), PB *n* (15.0°), PB *l* (23.6°), to PBs associated with coils with higher rmsda (namely PBs *h*, *g*, *p* for 50.0°, 52.6° and 52.6°, resp.) [72]. PBs are very efficient in tasks such as protein superimpositions [73,74] and MD analyses [75,76,77]. PB assignment was carried out for every residue of ECD1 using the PBxplore tool [41], available at GitHub (https://github.com/pierrepo/PBxplore. accessed on 23 February 2023). From this description, we have used a recognized measure that helps in quantifying the flexibility of each amino acid, called *N_eq_* (*N_eq_* stands for equivalent number of PBs) [40]. *N_eq_* is a statistical measurement similar to entropy and represents the average number of PBs a residue at a given position can adopt. The *N_eq_* is calculated as follows [40]:

$$N_{eq}=exp\left(-\sum_{x=1}^{16}f_{x}\;ln{\;f}_{x}\right)$$

where *f_x_* is the frequency of PB at the position (*x*). The *N_eq_* value can vary between 1 and 16. The *N_eq_* value of 1 means that only one type of PB is observed at a given position, meaning that the residue maintains its local 3D structure throughout the MD simulations, while a value of 16 indicates that the local 3D structure of a residue varies considerably in simulation time.

The PBxplore tool allows the assignment of PBs, the calculation of the *N_eq_* and the creation of sequence logos for PBs (WebLogo) [78], and was used recently to analyze intrinsically disordered proteins [79,80].

### 4.5. Analyses with Collective Variables for Secondary and Tertiary Structures 

These collective variables are described in detail in [50] and are designed to evaluate, by a continuous function of the coordinates, the fraction of secondary structure elements (α-helix, parallel β-sheets and antiparallel β-sheets) contained in a protein structure. For instance, for the antiparallel β-sheets, the variable counts how many pairs of three residues fragments in a given protein structure adopt the correct β-conformation, measured by the RMSD from an ideal block of antiparallel β-sheets formed by a pair of three residues. These variables were extensively employed for both structural analysis and enhanced sampling simulations in the case of folded, misfolded and disordered proteins [81,82,83,84].

The collective variable *s*(*R*) reads:

$$s\left(R\right)=\sum_{j}\frac{1-{\left(D/D_{0}\right)}^{8}}{1-{\left(D/D_{0}\right)}^{12}}$$

where *R* represents the set of all the atomic coordinates of the protein, *D* is the RMSD deviation between the ideal (PDB-based) and actual coordinates of atoms N, C_α_, C, O, and C_β_ in a set of six residues. Such residues are consecutive in the case of an α-helix, while they form two segments of three consecutive residues in the case of β structures, and the sum runs over all possible such six residue sets *j* on the protein chain. The parameter *D*_0_ is set to 1 Å, so that each term in the sum represents a function of *D* smoothly decaying from one to zero for increasing RMSD deviation: the collective variable counts the number of sets of six residues, along the protein chain, similar to the ideal α-helix, antiparallel or parallel β-sheet secondary structure elements. The total value of the collective variables is proportional to the number of residues adopting α-helix or β-sheet structures, while it is not exactly the number of such residues (for more details see Figure S5).

The free-energy surface (FES) plots were conducted using Gnuplot [85]. In each plot, the regions corresponding to the local minima (regions in blue to dark blue color) were analyzed. For each region, the pool of all the corresponding ECD1 conformations was extracted from the full-length MD simulation. Selected conformations were clustered according to the RMSD of the backbone atoms and the representative conformations of the first five most populated clusters are shown in Figure 4C, Figure 5C, Figure 6C and Figure 7C. Furthermore, the percentage of extracted conformations with respect to all the MD simulations run conformations (40,000) was calculated. In addition, the probabilities of secondary structures formation were calculated for the pool of structures found in the local minima, presented in Figure 4A, Figure 5A, Figure 6A and Figure 7A. To calculate the probabilities for the formation of secondary structures, we used in-house awk scripts. As the input file to awk scripts, the output file of the PBxplore tool, namely the file.PB.fasta was used, where the PBs are assigned for each residue in the protein chain. Our script reads the PB assigned sequence for each frame in the pool and prints the fraction of frames with a specific pattern. The patterns were defined as follows: (i) for an α-helix “mmmm” sequence in a range of residues 18–29 and (ii) for a β-strand “ddd” sequence anywhere in the ECD1 chain. Probabilities for different combination of these patterns were then calculated and are presented in Figure 4B, Figure 5B, Figure 6B and Figure 7B, as well as in Table S1.

### 4.6. Analyses

The analyses were conducted using the in-house Python programming language v.2.7.10 [86], and R software v.3.3.3 [87], while 3D visualization was performed using MacPyMOL software v.1.7.2.2 [88,89]. VMD was also extensively used [52]. Solvent accessibility was evaluated with NACESS software [47]. Secondary structures were assigned using DSSP 2.1 [90] and STRIDE software 1995 [51].

## 5. Conclusions

Inspection of the MD trajectories indicates that most of the ECD1 chain is disordered. More in detail, the lowest regions in the free-energy landscapes that we analyzed correspond to globular conformations with one α-helix at the position between Ser18 and Ser29, that is in many cases combined with an antiparallel β-sheet. The corresponding β-strands are most of the time formed at the positions 1–20 and 40–60. We also observed globular conformations with only β-sheet(s), however, they are thermodynamically less favored than conformations with an α-helix. We noticed that the amount of α-helices is much higher with respect to the amount of β-sheets (see the comparison between the values on the horizontal axes of different FES plots for the dark blue regions, Figure 4A, Figure 5A and Figure 6A). This result is in accordance with the PB analyses, which showed the highest probability for an α-helix formation (Figure 3, blue arrow). In addition, the α-helix is always formed between residues Ser18–Ser29, again corresponding well to the region identified by the PB analyses. Importantly, this observation is in perfect agreement with the experimental data, where the only part of the ECD1 that could be successfully crystallized was the α-helix at the position Phe22–Tyr30 [35], thus validating our approach and results. This work complements the recently proposed molecular dynamics simulations and experiments performed on the sulfated DARC peptide, showing that a sulfate on tyrosine 41 binds to a charged pocket on *Pv*DBP-RII [91], which is important in regards to the difficulty in designing a *P. vivax* vaccine [92,93,94].

Until now, a complete DARC 3D structure could not be obtained, despite different trials. Our results fill the gap in the structure, and molecular modeling groups can use this set of conformations in order to provide a complete DARC model, unifying the transmembrane part and the ECD1 domain. Docking and MD simulation studies on such DARC models could be carried out with different ligands, chemokines or Duffy binding protein domains for different *Plasmodium* (*P. vivax*, *P. knowlesi* and *P. cynomolgi*). Such in silico studies can help to understand the interactions between DARC and different ligands, which can in turn help to guide experiments for example mutational studies or structural studies, and can, finally, help to guide drug design. Our results could help to design potential vaccines, as they underline some interesting local conformations within disordered regions.

In a similar way, studying in parallel in silico *P. vivax* and *P. cynomolgi*, comparing their interactions with DARC, could be very useful to guide experimental studies on both *Plasmodium*. *P. vivax* is very challenging to cultivate in laboratory conditions, while *P. cynomolgi*, the closest living relative of *P. vivax,* is not. Such studies could help to advance the discovery of better therapies for *P. vivax* malaria.

## Data Availability

Various scripts and MD simulation files are available on reasonable request from the corresponding author (A.K.).

## Supplementary Materials

The following supporting information can be downloaded at: https://www.mdpi.com/article/10.3390/ijms241713280/s1. Reference [95] is cited in the supplementary materials.
