# Supplementary material for: Structural Space of the Duffy Antigen/Receptor for Chemokines’ Intrinsically Disordered Ectodomain 1 Explored by Temperature Replica-Exchange Molecular Dynamics Simulations
Source: Int J Mol Sci. 2023 Aug 26;24(17):13280. doi: 10.3390/ijms241713280 (PMC10488288; doi:10.3390/ijms241713280)
Supplement: Supplementary file 1 [file ijms-24-13280-s001.zip › ijms-2554652-supplementary/_RO_IJMS_Kranjc_SI.pdf]

SUPPLEMENTARY INFORMATION

Structural space of DARC's intrinsically disordered Ectodomain 1 explored by T-REMD simulations

Agata Kranjc [1,2,3,*], Tarun Jairaj Narwani [1,2], Sophie S. Abby [4] & Alexandre G. de Brevern [1,2,*]

[1] Université Paris Cité and Université des Antilles and Université de la Réunion, Biologie Intégrée du Globule Rouge UMR_S1134, DSIMB team, Inserm, F-75014 Paris, France; tjrnarwani@gmail.com, alexandre.debrevern@univ-paris-diderot.fr

[2] Institut National de la Transfusion Sanguine (INTS), F-75015 Paris, France

[3] Institute of Neuroscience and Medicine (INM-9)/Institute for Advanced Simulation (IAS-5), Forschungszentrum Jülich, D-52425 Jülich, Germany: a.kranjc.pietrucci@fz-juelich.de

[4] Univ. Grenoble Alpes, CNRS, UMR 5525, VetAgro Sup, Grenoble INP, TIMC, F-38000 Grenoble, France; sophie.abby@univ-grenoble-alpes.fr

* Correspondence: a.kranjc.pietrucci@fz-juelich.de (A.K.); alexandre.debrevern@univparis-diderot.fr (A.G.d.B.); Tel.: +33-040-4800

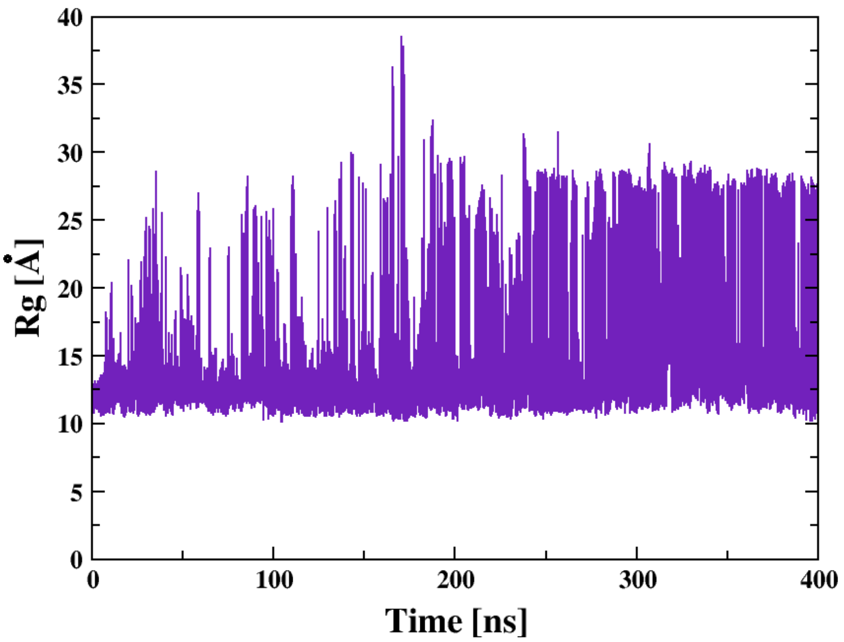

Figure S1. The radius of gyration variations (Å) of DARC ECD1 during MD simulations.

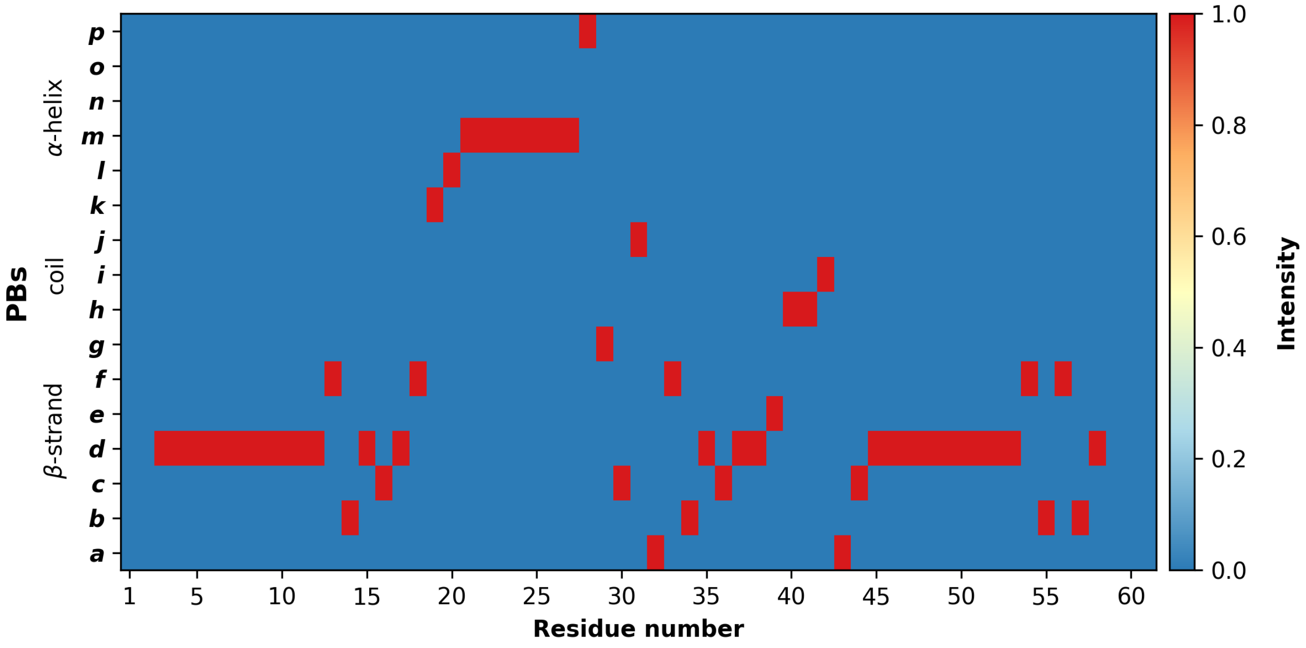

Figure S2. *Protein Blocks (PB) map for the cluster representative conformation 4 in region I of the* FES plot showing the amount of antiparallel β–sheets vs. radius of gyration (see Figure 5). The PB map clearly shows that residues at the positions 3-12 and 45-54 adopt local conformations corresponding to β-strand, while DSSP method implemented in VMD shows only coiled conformation.

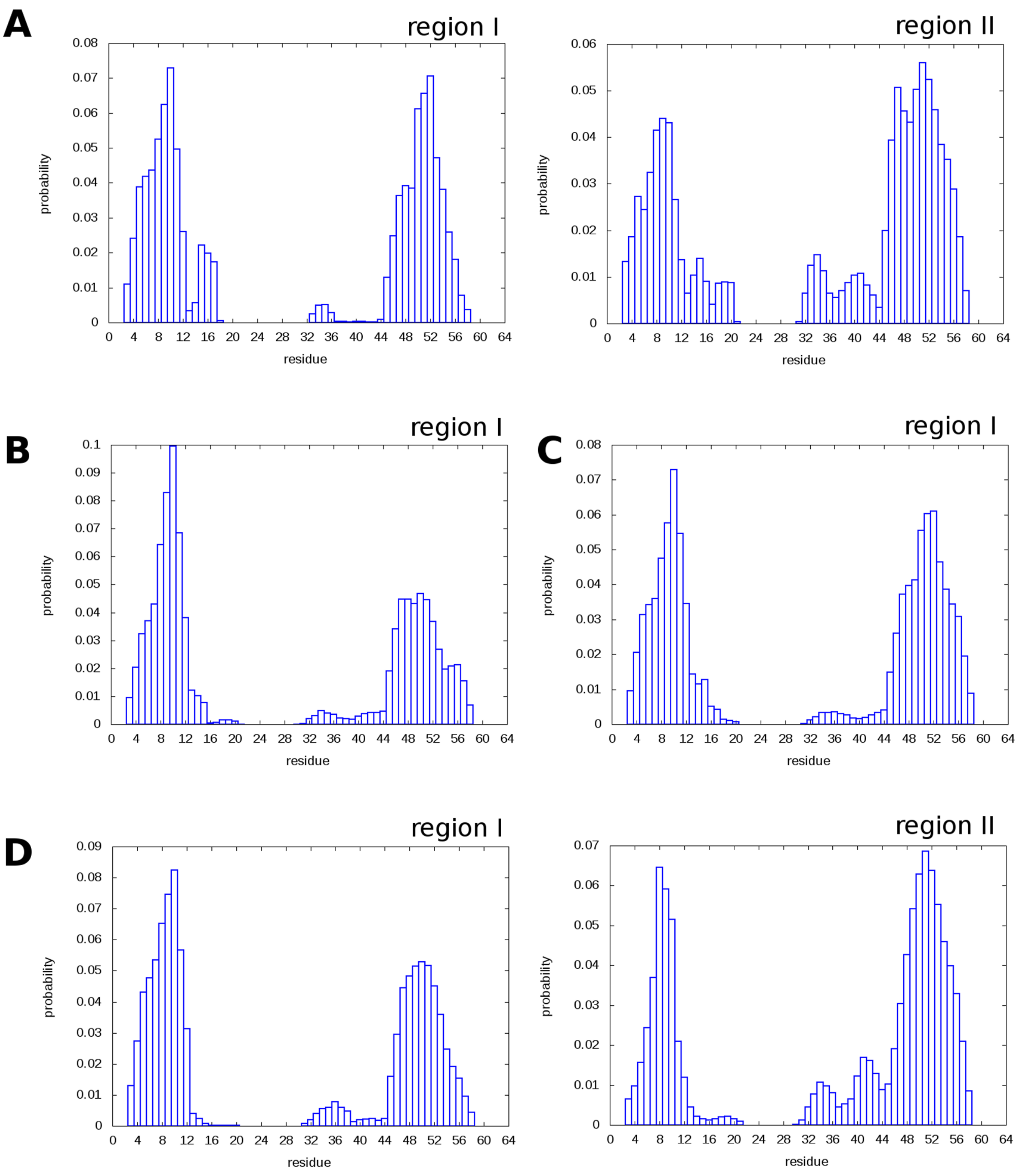

Figure S3. *Local probability of β strand formation when it co-exists with the α helix at the position 18-29.* For the ECD1 structures found in the local minima (regions I-IV) in the FES plot representing: **A**. amount of alpha *vs* Radius of gyration (Rg) (Figure 4); **B**. amount of antiparallel beta *vs* Rg (Figure 5); **C.** amount of parallel β *vs* Rg (Figure 6); **D.** amount of α *vs* antiparallel β (Figure 7). Note that regions III and IV in the Figures 4 and 7 have zero amount of the α-helix; therefore α and β cannot co-exist. To see the local probability for only β formation, see Figure S5.

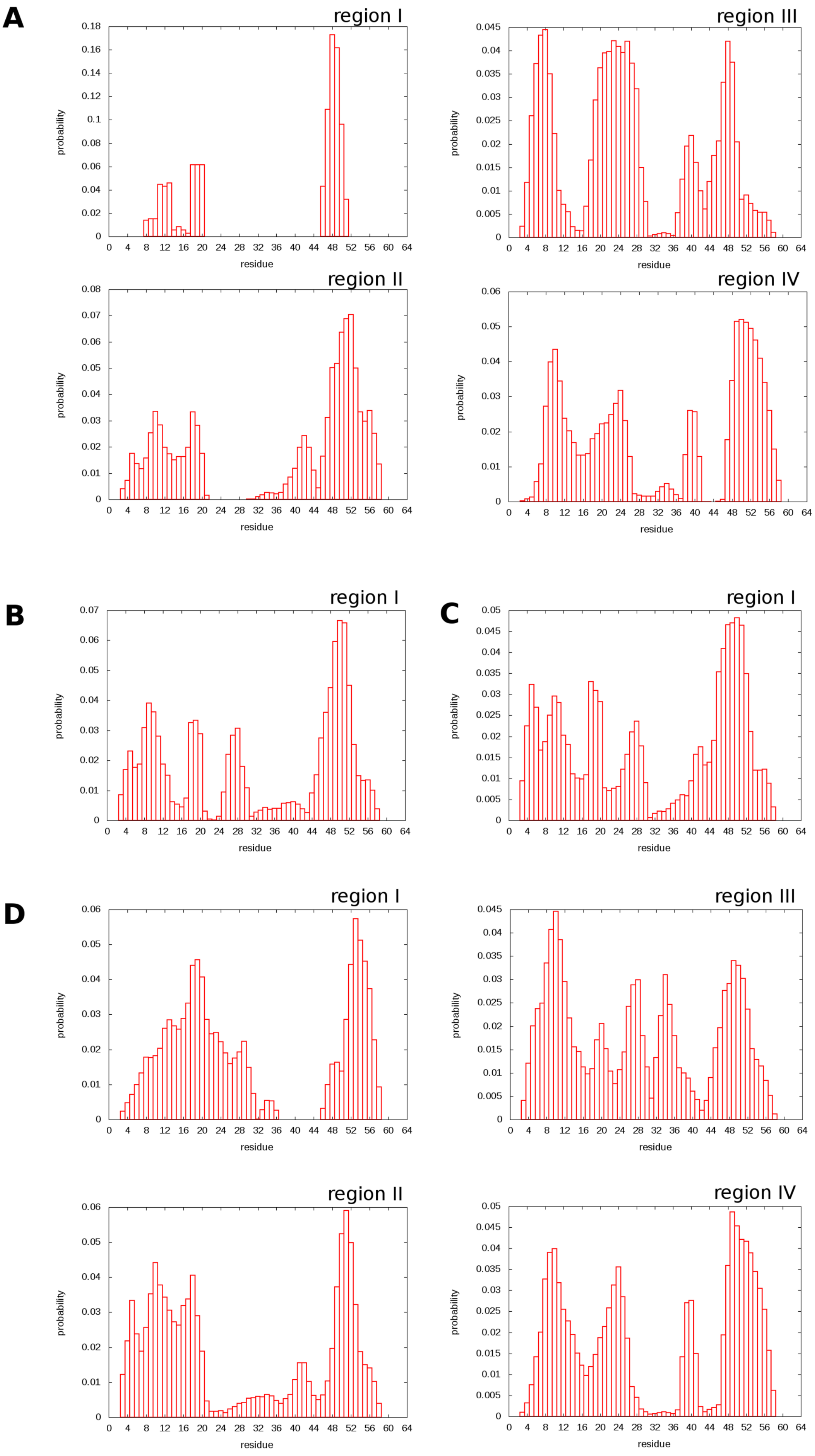

A
region I
region III
region II
region IV
B
region I
C
region I
D
region I
region III
region II
region IV
probability
residue

Figure S4. *Local probability of forming beta strand within the ECD1 sequence when alpha helix at the position 18-29 is absent.* For the ECD1 structures found in the local minima (regions I-IV) in the FES plot representing: **A**. amount of α vs Radius of gyration (Rg) (Figure 4); **B**. amount of antiparallel β vs Rg (Figure 5); **C.** amount of parallel β-sheet *vs* Rg (Figure 6); **D.** amount of α vs antiparallel β-sheet (Figure 7).

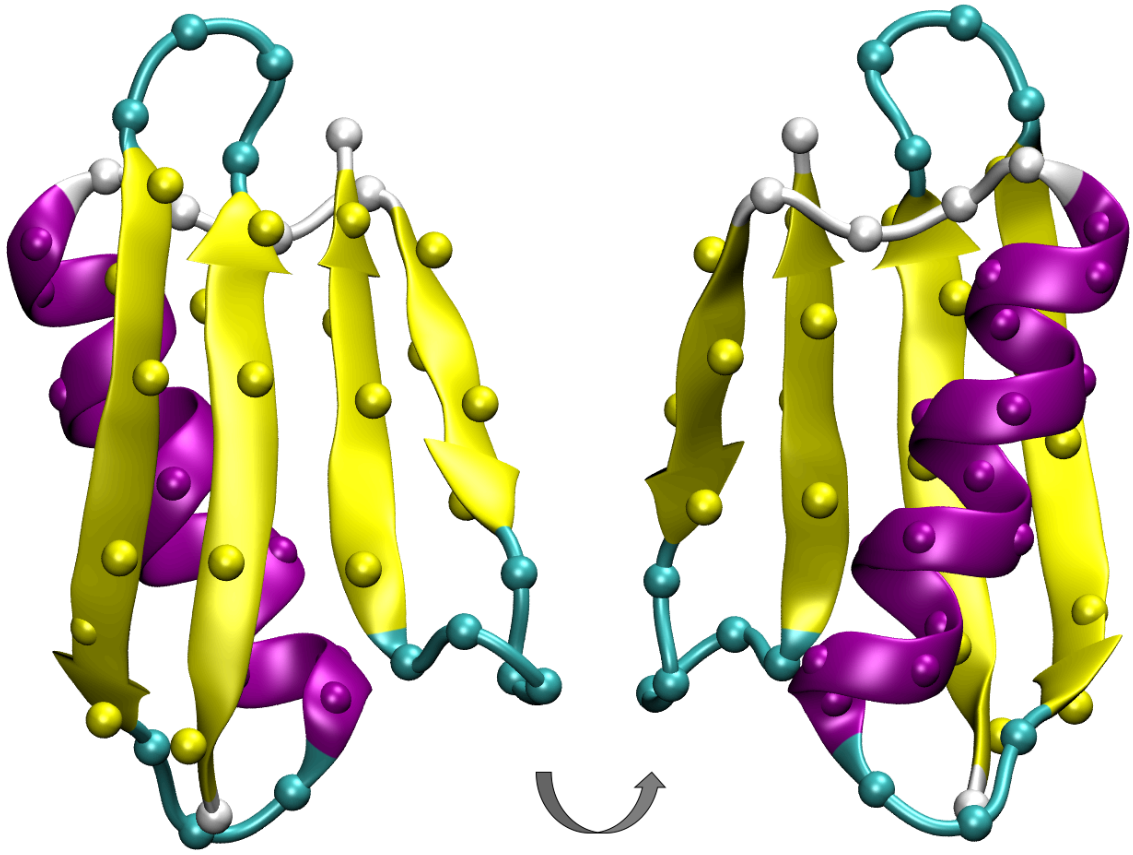

Figure S5. *The example of values of the collective variables for an ideal α–helix or β–sheet structures* calculated on the case of GB1 protein (PDB id: 2J52 [1]. The a collective variable for the α–helix of GB1 (violet color) is of 12.8, whereas for a *parallel* β–sheet and the two *antiparallel* β–sheets (yellow color) connected by β–hairpins, the values of the β–variables are 6.1 and 9.0, respectively. The protein is shown from two different sides for clarity reasons. Cα atoms are shown as balls.

Table S1. *Fraction of the ECD1 conformations featuring secondary structures: an α–helix at position 18–29 and ß–strands*. The secondary structure analyses were done on the pool of structures found in the selected local minima appearing in the plots of the free energy surface (FES) analyses done for: (i) the amount of α–helices versus the radius of gyration (Rg), (ii) amount of antiparallel ß-sheet *vs* Rg, (iii) amount of parallel ß-sheet *vs* Rg and (iv) amount of α-helices vs antiparallel ß-sheets (Figures 4A –7 A in the main text, respectively). The total number of ECD1 structures found in each region is reported as well as the fraction of the structures that has: (i) α–helix at position 18–29 (ii) ß –strand formed anywhere in the sequence (iii) α–helix at position 18–29 and at the same time ß–strands; the range of residue positions that have the highest probability to form ß–strands are reported. In addition, we show the fraction of structures having beta strands for different ranges of the ECD1 residues positions (iv) α–helix at position 18–29 absent and only ß–strands are present; the range of residue positions that have the highest probability to form ß –strands are reported. In addition, we show the fraction of structures featuring beta strands for different ranges of the ECD1 residues positions.

An example: In the first local minima – the Region I – of the plot "Amount of alpha vs Rg" we found 1741 ECD1 conformations. 73% of these structures have an alpha helix at the position 18–29 and 76% have ß–strand(s) anywhere in the ECD1 chain. 71% of these structures have alpha helix at the position 18–29 and at the same time ß–strands, that form with the highest probability at the N– and C–terminal part of the ECD1 chain, in the range of residues 1–20 and 40–60 forming a ß–sheet. It is shown as well the fraction of the ECD1 conformations having ß–strands simultaneously at different residues ranges: 1–20 & 30–40, 30–40 & 40–60, 1–20 & 40–60 and 1–20, 30–40 & 40–60. Finally, 5% of all structures in this minimum feature only ß–strands, that forms with the highest probability at the ECD1 residues 1–20 and 40–60. And looking more in a detail, we see that in this case ß–strands are never formed simultaneously in other of the ECD1.

			ALPHA POSITION 18–29	BETA ANYWHERE	COEXISTENCE of ALPHA+BETA		ONLY BETA without alpha	
	**LOCAL MINIMUM**	**TOTAL N° of STRUCTURES**	**Fraction**	**Fraction**	**Fraction**	**Beta position**	**Fraction**	**Beta position**
**Amount of alpha vs Rg**	**Region I**	1741	0.73	0.76	0.71	1–20, 40–60	0.05	1–20, 40–60
					**Beta position**	**Fraction**	**Beta position**	**Fraction**
					1–20, 30–40	0.003	1–20, 30–40	0
					30–40, 40–60	0.04	30–40, 40–60	0
					1–20, 40–60	0.57	1–20, 40–60	0.30
					1–20, 30–40, 40–60	0.002	1–20, 30–40, 40–60	0
	**Region II**	1749	0.51	0.83	0.44	1–20, 40–60	0.40	1–20, 40–60
					**Beta position**	**Fraction**	**Beta position**	**Fraction**
					1–20, 30–40	0.15	1–20, 30–40	0.04
					30–40, 40–60	0.15	30–40, 40–60	0.02
					1–20, 40–60	0.54	1–20, 40–60	0.51
					1–20, 30–40, 40–60	0.09	1–20, 30–40, 40–60	0.02
	**Region III**	448	0	1	0	——	1	1–20, 40–60
					**Beta position**	**Fraction**	**Beta position**	**Fraction**
					1–20, 30–40	0	1–20, 30–40	0.33

**Amount of alpha vs Rg**					30–40, 40–60	0	30–40, 40–60	0.31
					1–20, 40–60	0	1–20, 40–60	0.90
					1–20, 30–40, 40–60	0	1–20, 30–40, 40–60	0.31
	**Region IV**	952	0	1	0	——	1	1–20, 40–60
					**Beta position**	**Fraction**	**Beta position**	**Fraction**
					1–20, 30–40	0	1–20, 30–40	0.82
					30–40, 40–60	0	30–40, 40–60	0.82
					1–20, 40–60	0	1–20, 40–60	1.00
					1–20, 30–40, 40–60	0	1–20, 30–40, 40–60	0.82
**Amount of antiparallel beta vs Rg**	**Region I**	7111	0.78	0.86	0.70	1–20, 40–60	0.15	1–20, 40–60
					**Beta position**	**Fraction**	**Beta position**	**Fraction**
					1–20, 30–40	0.06	1–20, 3 [illegible]	0.03
					30–40, 40–60	0.07	30–40, 40–60	0.01
					1–20, 40–60	0.42	1–20, 40–60	0.38
					1–20, 30–40, 40–60	0.05	1–20, 30–40, 40–60	0.01

			ALPHA POSITION 18–29	BETA ANYWHERE	COEXISTENCE of ALPHA+BETA		ONLY BETA without alpha	
	**LOCAL MINIMUM**	**TOTAL N° of STRUCTURES**	**Fraction**	**Fraction**	**Fraction**	**Beta position**	**Fraction**	**Beta position**
**Amount of parallel beta vs Rg**	**Region I**	11218	0.82	0.78	0.66	1–20, 40–60	0.12	1–20, 40–60
					**Beta position**	**Fraction**	**Beta position**	**Fraction**
					1–20, 30–40	0.02	1–20, 30–40	0.03
					30–40, 40–60	0.03	30–40, 40–60	0.02
					1–20, 40–60	0.50	1–20, 40–60	0.39
					1–20, 30–40, 40–60	0.01	1–20, 30–40, 40–60	0.02
**Amount of alpha vs amount of antiparallel beta**	**Region I**	3453	0.84	0.90	0.82	1–20, 40–60	0.08	1–20, 40–60
					**Beta position**	**Fraction**	**Beta position**	**Fraction**
					1–20, 30–40	0.15	1–20, 30–40	0.02
					30–40, 40–60	0.15	30–40, 40–60	0.07
					1–20, 40–60	0.70	1–20, 40–60	0.58
					1–20, 30–40, 40–60	0.14	1–20, 30–40, 40–60	0.02
	**Region II**	1171	0.38	0.77	0.25	1–20, 40–60	0.52	1–20, 40–60
					**Beta position**	**Fraction**	**Beta position**	**Fraction**
					1–20, 30–40	0.06	1–20, 30–40	0.07

**Amount of alpha vs amount of antiparallel beta**					30–40, 40–60	0.11	30–40, 40–60	0.04
					1–20, 40–60	0.50	1–20, 40–60	0.53
					1–20, 30–40, 40–60	0.05	1–20, 30–40, 40–60	0.03
	**Region III**	336	0	1	0	——	**1**	1–20, 40–60
					**Beta position**	**Fraction**	**Beta position**	**Fraction**
					1–20, 30–40	0	1–20, 30–40	0.49
					30–40, 40–60	0	30–40, 40–60	0.47
					1–20, 40–60	0	1–20, 40–60	0.91
					1–20, 30–40, 40–60	0	1–20, 30–40, 40–60	0.47
	**Region IV**	813	0	1	0	——	1	8–11 ; 22–25 ; 49–52
					**Beta position**	**Fraction**	**Beta position**	**Fraction**
					1–20, 30–40	0	1–20, 30–40	0.73
					30–40, 40–60	0	30–40, 40–60	0.73
					1–20, 40–60	0	1–20, 40–60	0.99
					1–20, 30–40, 40–60	0	1–20, 30–40, 40–60	0.73

Data S1. *PDB files of the ECD1 representative structures shown in Figure 4.*

Data S2. *PDB files of the ECD1 representative structures shown in Figure 5.*

Data S3. *PDB files of the ECD1 representative structures shown in Figure 6.*

Data S4. *PDB files of the ECD1 representative structures shown in Figure 7.*

Reference

1. Wilton, D.J.; Tunnicliffe, R.B.; Kamatari, Y.O.; Akasaka, K.; Williamson, M.P. Pressure-induced changes in the solution structure of the gb1 domain of protein g. *Proteins* **2008**, *71*, 1432-1440.
